# Making the Transition from Student to Resident: A Method to Individualize a PGY1 Program

**DOI:** 10.3390/pharmacy4040031

**Published:** 2016-10-20

**Authors:** Amy N. Thompson, Jean Nappi, Brian McKinzie, Jason Haney, Nicole Pilch

**Affiliations:** 1 College of Pharmacy, Medical University of South Carolina, Charleston, SC 29425, USA; nappijm@musc.edu (J.N.); haney@musc.edu (J.H.); 2 Department of Pharmacy Services, Medical University of South Carolina, Charleston, SC 29425, USA; mckinzi@musc.edu; 3 Transplant ICCE, Medical University of South Carolina, Charleston, SC 29425, USA; weimert@musc.edu

**Keywords:** customized plan, residency, examination

## Abstract

A Postgraduate Year One (PGY1) resident’s concerns, limitations, and strengths may be self-identified early in the residency year but are reliant on self-awareness and insight. Program directors commonly find difficulty in identifying a resident’s specific knowledge deficits at the beginning of the program. A standardized resident examination can identify limitations early in training and these results can be incorporated into a tailored resident development plan. A total of sixty-two PGY1 residents completed the examination pre- and post-training over a five-year timespan. Scores increased in most core disciplines in each of the five years, indicating an overall improvement in resident knowledge throughout their PGY1 year. The approach of utilizing the scores for the resident’s individualized plan allows for customization to ensure that the resident addresses knowledge gaps where necessary.

## 1. Introduction

The transition from student to resident can be a challenge for both the new resident and the residency program director (RPD). Many residents struggle with balancing clinical obligations and other residency requirements. The majority of concerns, limitations, and strengths may be revealed during a discussion with the resident, however sometimes it is difficult to pinpoint specific knowledge deficits at the beginning of the program. The ASHP Accreditation Standard for Postgraduate Year One (PGY1) programs, states that the residents’ development plan should be designed to address each resident’s unique learning needs, and include both incoming strengths and weaknesses [1]. To our knowledge, there is currently no literature assessing resident readiness prior to a PGY1 residency or describing how to appropriately individualize the resident’s development plan. Currently there is no standard for clinical knowledge assessment upon exit from pharmacy school. In an effort to try to identify specific limitations early in the year, we began administering an examination covering a broad range of topics given during orientation in July of the residency year. This examination was used to help the resident and RPD identify strengths and weaknesses during the orientation month and create an individualized development plan. At the end of the residency year, the residents took the same exam and their results compared (pre- and post-residency experience). The aim of this study it to describe the process of incorporating examination results into development plans for 62 residents given the examination over the last five years.

## 2. Methods

A bank of questions was developed by preceptors from each of the core rotation disciplines: critical care (including solid organ transplantation and nutrition), drug information, internal medicine (including ambulatory care and oncology), operations, pediatrics, practice management, and psychiatry (Table 1). Full details on the exam were previously published [2]. Questions were assigned a difficulty rating: graduating pharmacy student (level 1), up to clinical specialist (level 3). Examples of the three levels of difficulty can be found in Appendix A. The majority of questions did not change from year to year; however, if a particular question was routinely missed on pre- and post-examination or if practice guidelines changed, the question was exchanged for a question in the same practice area with a similar degree in difficulty. The examination, consisting of 50 questions, was structured to cover diverse patient populations and disease states with varying complexities. Five different classes of residents were assessed over a five-year time frame, July 2011 to June 2016.

This examination was given to each resident at the beginning of their orientation month and during the last month of their PGY1 residency year. The results were provided to the residency advisors and residents to help develop their customized plans for the year. At the end of each year the exam was assessed; questions that involved diseases with new standards of care or medications no longer available or which performed poorly were replaced with questions of the same level of difficulty and discipline. A Student’s *t*-Test was utilized to compare differences in overall score, as well as changes in knowledge in the three levels and seven disciplines in the pre- and post-exams (Microsoft^®^ Excel^®^ 2010). A *p*-value less than 0.05 was considered statistically significant. This project was approved as exempt from IRB approval.

## 3. Results

A total of sixty-two PGY1 residents completed the examination pre- and post-training over a five-year timespan: nine residents in year one, fourteen in year two, and thirteen in years three, four, and five. The results of each “pre-residency training” examination were incorporated into the resident’s development plan (Appendix B). The approach of utilizing the scores within the customized plan aids in identifying knowledge gaps with the individual, and allowing for customization to ensure that the resident sees growth where necessary. Scores increased in most core disciplines in each of the five years, indicating an overall improvement in resident knowledge throughout their PGY1 year.

## 4. Discussion

The use of a standardized resident examination was first used within our institution and results are described in detail elsewhere [2]. We previously described the utility of this examination to quantify changes in resident knowledge throughout the year as well as provide insight into potential targeted areas for improvement in the residency program. Likewise, the results of this examination are useful for detecting potential knowledge deficits of the resident and developing a customized PGY1 learning plan. Identified early in the year, this may help reduce unnecessary stress for both the resident and program director. It has been reported that 6%–15% of pharmacy students have some form of academic deficiency during their training [3]. Currently there is not published data on the percentage of pharmacy residents reporting difficulty or needing intervention during their PGY1 year. However, our medical colleagues have noted that approximately 8%–15% of residents struggle during their residency [3]. Additionally, unlike pharmacy school, most pharmacy residency programs do not have structured assistance programs designed to help the resident [4].

Given that our exam was addressing core disciplines, we were able to hone in on particular areas that may need further development for a resident. For example, a resident who did well in internal medicine but struggled with critical care may benefit from having a critical care experience earlier in the year or an additional rotation in that area to help increase their knowledge base. Alternatively, utilizing the areas of strength may help a resident with the selection of a PGY2 specialty residency. This too would be beneficial early in the year to allow for more experience with that specialty prior to the annual recruitment process for PGY2 programs.

There are several limitations to our study. First, a larger proportion of questions focused on the medicine and critical care core disciplines which parallel the resident experiences in our PGY1 residency program. Accordingly, exam performance in other core disciplines may not accurately reflect each resident’s knowledge in that area. We did not account for testing bias, including the possibility of score inflation from repeated exposure to exam questions. Studies of higher-stakes examinations in medical training, however, have shown that prior exposure does not significantly improve performance [5,6]. Finally, we did not assess if this type of exam correlated with self- or preceptor-assessed performance.

The medical profession utilizes standardized examinations throughout school and during residency training to ensure that certain minimum knowledge requirements are met. Although licensure exams are required in the United States, pharmacy has lagged behind in ensuring that there is a minimum level of competency obtained from clinical training by allowing board exams to be optional. A significant amount of literature exists surrounding the use of examinations to facilitate medical resident selection however they do not predict performance in the residency [7]. Rather exam performance did predict first time pass rates of board exams following residency training [7]. As such we suggest that further investigation of and implementation of an exam requirement at the end or the beginning of pharmacy residency training will facilitate standardization of minimum knowledge requirements and ensure that the resident has met a standard not currently available. Additional studies are necessary to determine the benefits of standardized exams in pharmacy residency training.

Although we expected to see improvement in exam scores once residents completed their PGY1 year, we feel that giving a comprehensive written examination at the beginning of the residency year helps both the resident and program director construct an individualized, comprehensive development plan which helps with program planning. Being able to identify specific strengths and weaknesses at the beginning of the year can help make the year more successful for the resident by tailoring it to better fit their specific needs. Future research is needed to determine the role of post-graduate residency training and competency testing. This leads to a final question: with advanced pharmacy degrees and a high level of clinical practice should the pharmacy community embrace the medical competency model with testing following each step of training to ensure that the product produced meets minimum standards?

## Figures and Tables

**Table 1:** Postgraduate Year One (PGY1) Resident Performance on a Standardized Examination from 2011 to 2016 (*n* = 62).

	Number of Questions	Exam Scores (% Correct)		*p*-Value
		Pre-PGY1 ^a^	Post-PGY1 ^b^	
Overall	50	64.2	73.0	<0.001
Level 1	27	68.3	75.7	<0.001
Level 2	19	64.6	75.7	<0.001
Level 3	4	44.0	47.5	NS
**Core Disciplines**				
Critical Care	14	58.0	68.7	<0.001
Drug Information	4	70.6	72.6	NS
Internal Medicine	17	56.5	69.2	<0.001
Operations	6	85.3	89.9	NS
Pediatrics	3	57.1	59.2	NS
Practice Management	2	89.5	79.8	0.013
Psychiatry	4	73.0	84.3	<0.001

^a^ Pre-PGY1, during the first month of PGY1 residency; ^b^ Post-PGY1, during the last month of PGY1 residency.

## Appendix A. Example Questions

### Appendix A1. Medicine, Level 1

1. A 55-year-old Caucasian female with a past medical history significant for hypertension, type 2 diabetes, stage 3 chronic kidney disease, and gastroesophageal reflux disease (GERD) presents to your clinic today complaining of unilateral left lower extremity swelling and pain. An ultrasound is ordered and a deep venous thrombosis (DVT) is diagnosed. The physician plans to initiate therapy with enoxaparin 80 mg SC daily and warfarin 5 mg PO daily, with PT/INR follow-up in 3 days.
  *Weight*: 82 kg *Height*: 5’9’’
  *Labs*: SCr 2.9 mg/dL; AST 25 IU/L; ALT 18IU/L; Alk Phos 45 IU/L; INR 0.8
  Which of the following is the most appropriate plan at this time?
  - Increase enoxaparin to 80 mg SC BID as this is the appropriate dosing for treatment of DVT. Continue warfarin 5 mg PO daily.
  - Decrease enoxaparin to 30 mg SC daily as this is the appropriate dosing for treatment of DVT. Continue warfarin 5 mg PO daily.
  - Continue enoxaparin 80 mg SC daily, order anti-factor Xa serum concentration 12 h after dose. Continue warfarin 5 mg PO daily.
  - Continue enoxaparin 80 mg SC daily, order anti-factor Xa serum concentration 4 h after dose. Continue warfarin 5 mg PO daily.

### Appendix A2. Drug Information, Level 2

2. You are on rotation in the Drug Information Center and receive a call from the Compounding Pharmacy. The Rutledge Tower Pharmacy has received a prescription for omeprazole suspension. Before preparing, the pharmacist in the Compounding Pharmacy needs a formulation with stability data. Which PubMed search is the *BEST* choice for finding relevant articles?
  - Omeprazole AND suspension = 109 results
  - Omeprazole [MeSH] AND (drug compounding [MeSH] OR pharmaceutical preparations [MeSH]) = 37 results
  - Omeprazole [MeSH] AND drug compounding [MeSH] AND suspension [MeSH] = 3 results
  - Omeprazole [MeSH] OR (suspension [MeSH] AND drug compounding [MeSH] AND pharmaceutical preparations [MeSH]) = 7755 results

### Appendix A3. Critical Care-Nutrition, Level 3

3. A 58-year-old morbidly obese Caucasian male in the Medical Surgical ICU is POD 7 from small bowel resection due to bowel ischemia. The patient is currently intubated and NPO except medications via NG tube. The team would like to start trophic feeds but is awaiting the surgery team’s approval. On rounds, the team consults you to initiate PN on this patient until the patient is at his goal tube feeding rate.
  *Weight:* 190 kg *Height:* 68 in
  *Past medical history:* hypertension, diabetes type II, obstructive sleep apnea, osteoarthritis, acute kidney injury
  *Current pertinent medications*: hydromorphone infusion (1 mg/h); propofol infusion (35 micrograms/kg/min); famotidine 20 mg IV Q24H; heparin 7500 units SC Q8H; insulin infusion (2 units/h); aluminum hydroxide 30 mL NG Q8H
  *Pertinent labs*: Na 140 mEq/L; Cl 108 mEq/L; K 4.0 mEq/L; CO_2_ 18 mEq/L; BUN 60 mg/dL; SCr 4.0 mg/dL; ionized Ca 1.15 mmol/L; Mg 2.0 mg/dL; Phos 3.5 mg/dL
  Which of the following groups of macronutrients is the most appropriate for the initial PN for this patient?
  - Total calories 4182 kcal, Protein 285 gm, CHO 536 gm, Lipids 122 gm
  - Total calories 995 kcal, Protein 100 gm, CHO 175 gm, Lipids 0 gm
  - Total calories 1478 kcal, Protein 140 gm, CHO 270 gm, Lipids 0 gm
  - Total calories 1501 kcal, Protein 100 gm, CHO 162 gm, Lipids 55 gm

## Appendix B. Examples of How Examination Is Used in Planning of Resident Development

### Appendix B1. Resident #1

**Table:**

Entering Characteristics	Initial Plan/Changes to the Program/Residency Structure
**Areas for Improvement (list):** - Communication (Verbal and Written) - Leadership - Teaching/Precepting - Pharmacotherapy Knowledge (Psych, BMT, Renal Insufficiency, Medical Emergencies	- Will co-precept a P4 in August (teaching/precepting) - Will quickly be immersed into the team environment on rounds and will speak up to provide recommendations (verbal) - Will produce manuscripts suitable for publication (written/MUE/project) - Will actively request topic discussions on rotation to increase knowledge in pharmacotherapy areas of weakness (Knowledge) - Results from Entrance Exam indicate: - 64% overall score - Areas of weakness: Critical care, Medicine, Pediatrics, Drug information, Operations (all rotations she will have and be exposed to) - Did not miss a single question in Management or Psychiatry
**Interests (list):** - Critical Care - Hematology/Oncology	- Will be exposed to both Critical Care (STICU in August) and Hematology/Oncology (September) prior to Midyear

### Appendix B2. Resident #2

**Table:**

Entering Characteristics	Initial Plan/Changes to the Program/Residency Structure
**Areas for Improvement:** - High expectations of myself and others - Based on baseline competency exam, areas of focus this year should include drug information, management and pediatrics - Management	- I hope to continue to work on setting more realistic goals for others and myself by being flexible and communicating effectively. - Improve baseline competencies. To improve my drug information skills, I will complete my drug information rotation and take part in call for four weeks over the year. To improve my management skills, I will complete a management rotation, and practice management skills on my MUE and longitudinal project. Finally, I will improve my pediatric skills by completing a general pediatric rotation, staffing in the children’s hospital over the first year of residency and completing an integrated practice rotation in pediatrics. - I will complete a management rotation as part of the general requirements for the residency. Additionally, through my staffing and teaching opportunities, I will learn how to lead and manage a team.
**Interests:** - Internal Medicine - Cardiology - Critical Care	- Confirm interests in internal medicine and cardiology through rotations during PGY1 year and explore interest in critical care during my PGY2 year.
